# Determination of *β*-Agonist Residues in Animal-Derived Food by a Liquid Chromatography-Tandem Mass Spectrometric Method Combined with Molecularly Imprinted Stir Bar Sorptive Extraction

**DOI:** 10.1155/2018/9053561

**Published:** 2018-06-25

**Authors:** Jiwang Tang, Jianxiu Wang, Shuyun Shi, Shengqiang Hu, Liejiang Yuan

**Affiliations:** ^1^College of Chemistry and Chemical Engineering, Central South University, Changsha 410083, China; ^2^Hunan Testing Institute Product and Commodity Supervison, Changsha 410007, China

## Abstract

A novel clenbuterol molecularly imprinted polymer (MIP)-coated stir bar was prepared and applied to the determination of six *β*-agonists in animal-derived food. Characterization and various parameters affecting adsorption and desorption behaviours were investigated. The extraction capacities of clenbuterol, salbutamol, ractopamine, mabuterol, brombuterol, and terbutaline for MIP coating were 3.8, 2.9, 3.1, 3.5, 3.2, and 3.3 times higher, respectively, than those of the NIP coating, respectively. The method of MIP-coated SBSE coupled with HPLC-MS/MS was developed. The recoveries in pork and liver samples were 75.8–97.9% with RSD from 2.6 to 5.3%. Limits of detection (LODs) and limits of quantification (LOQs) were 0.05–0.15 *μ*g/kg and 0.10–0.30 *μ*g/kg, respectively. Good linearities were obtained for six *β*-agonists with correlation coefficients (*R* ^2^) higher than 0.994. These results indicated the superiority of the proposed method in the analysis of *β*-agonists in a complex matrix.

## 1. Introduction

*β*-adrenergic agonists are a kind of chemical synthesized from the benzyl ethanol amines. These compounds have been extensively used as growth promoters in livestock production, with advantages of increasing the animal's lean meat, improving feed efficiency, and reducing the number of days to market [1, 2]. Consumption of animal tissues with high *β*-adrenergic agonist content by humans leads to symptoms such as muscle tremor, muscle pain, nausea, and dizziness and can pose a serious threat to life [3]. Owing to illegal *β*-agonist use and its potential hazardous effects on human health, some countries have banned its use in farm animals [4]. The wide variety of these compounds and their extensive use in farm animals give rise to enormous challenges of daily supervision and routine monitoring for the government. It is therefore necessary to develop a validated method with characteristics of high throughput and high sensitivity for the detection and quantification of *β*-adrenergic agonists.

Recently, several analytical methods have been developed for the determination of *β*-agonist drugs in foodstuffs and animal urine [5–13]. The analytical methods are mainly based on two different immune analysis techniques [6, 7, 9, 11] and on the chromatographic technique [5, 8, 10, 12, 13]; both have advantages and drawbacks. Immune analysis techniques such as enzyme-linked immunosorbent assay (ELISA) exhibit the features of rapid and simple analysis but come with drawbacks of giving rise to false positives. Chromatographic techniques such as HPLC, GC-MS, and LC-MS/MS require expensive instrumentation and cumbersome sample pretreatments but have advantages of high accuracy and sensitivity. High-performance liquid chromatography-mass spectrometry/mass spectrometry (HPLC-MS/MS) is a feasible method that is commonly used due to its advantages such as sensitivity and accuracy [14]. For all mass spectrometry analytical procedures, the sample matrix effect is a major problem that influences the accuracy and precision of the testing results [15, 16]. Therefore, sample preparation is important and crucial, occupying nearly seventy percent of the total analysis time.

Research into sample preparation techniques has become a frontier of modern analytical chemistry, motivated by the demand for preparation methods with advantages of reduced or eliminated solvent, high selectivity, simplicity, speed, and so on. Traditional techniques such as liquid-liquid extraction (LLE) [17, 18], immune-affinity chromatography [19], and solid-phase extraction (SPE) [20, 21] are time-consuming and labour-intensive. The advanced and nonexhaustive techniques such as SPME (solid-phase microextraction) are solvent-free sample preparation techniques and combine sampling, analyte enrichment, and purification into one step, therefore substantially reducing the cost and total time of analysis [22]. The SPME contains various extraction concepts such as stirrers, vessels, coated fibres, and membranes [23]. Among these techniques, stir bar sorptive extraction (SBSE), first introduced in 1999 by Baltussenetal [24], is an environmentally friendly sample preparation technique using a stir bar coated with polydimethylsiloxane (PDMS). However, SBSE with a PDMS-coated stir bar shows low selectivity and specificity for target analyte and is only suitable for the analysis of nonpolar and weakly polar compounds [25]. Molecularly imprinted polymer (MIP) coatings for SBSE were proposed to solve the problem of polar molecules and selectivity. MIPs are prepared by copolymerization of functional monomers and cross-linkers in the presence of template molecules (target analytes). Extraction of the template molecules leaves the recognition sites in the polymers with specific size, shape, and functional group complementarity to the original print molecule [26]. Thus, MIPs show selective recognition and high affinity to the template molecule and its structurally related compounds [27]. Moreover, the imprinted polymers are robust, stable, and resistant to a wide range of pH, temperature, and solvents [28, 29]. The MIP-SBSE technology that combines MIP with SBSE plays an important role in the aspects of removing or decreasing sample matrix effect. The combination of MIP-SBSE technology with liquid chromatography has also been reported [30–38]. However, the combination of MIP-SBSE technology with liquid chromatography-tandem mass spectrometry has been rarely reported.

The aim of this present work was to prepare an MIP coating on the glass stir bar and apply it to the selective SBSE of clenbuterol and its analogue compounds. The preparation parameters and extraction conditions, which affected the extraction performance of the stir bar, were studied in detail. The extraction capacity and selectivity of the clenbuterol MIP-coated stir bar were also evaluated. Then, a method of determination of structural-related *β*-agonists by MIP-SBSE followed by HPLC-MS/MS was developed. The proposed method was successfully applied to the analysis of *β*-agonists in animal-derived food.

## 2. Experimental Section

### 2.1. Reagents and Standards

Clenbuterol, salbutamol, ractopamine, mabuterol, brombuterol, and terbutaline were purchased from Dr. Ehrenstorfer GmbH (Augsburg, Germany). The functional monomer methacrylic acid (MAA) and the free radical initiator azoisobutyronitrile (AIBN) were supplied by Qiangsheng Chemical Co. Ltd. (Jiangsu, China). The cross-linking agent ethylene glycol dimethacrylate (EGDMA) was supplied by Aladdin Industrial Co. (Shanghai, China). The silane coupling agent KH-570 was purchased from Sinopharm Chemical Reagent Co. Ltd. (Shanghai, China). HPLC-grade methanol, toluene, and acetonitrile were purchased from Merck (Darmstadt, Germany). Water for HPLC-MS/MS was purified using a Milli-Q water purification system (Millipore, USA). Glass capillary (2 mm diameter, 100 mm length) was obtained from Guangzhou Fine Packaging Equipment Co., Ltd.

### 2.2. Apparatus

The coating surface of the stir bar was characterized by a VEGA3 scanning electron microscope (TESCAN, Czech Republic), and the composition of the coating was investigated using a Fourier transform infrared (FT-IR) spectrometer (Nicolet iS5, Thermo, USA). Measurements were taken using a TSQ Quantum Ultra triple quadrupole mass spectrometer (Thermo, USA) equipped with an electrospray ion source and coupled with an Accela liquid chromatography (Thermo, USA), equipped with a Hypersil GOLD (100 mm × 2.1 mm i. d., 3 *μ*m) column.

### 2.3. HPLC-MS/MS Conditions

The mobile phase consisted of mobile phase A (0.1% formic acid in water) and mobile phase B (acetonitrile) at the flow rate of 0.3 mL·min^−1^ using a gradient elution program. The solvents were degassed by an in-line vacuum degasser. The gradient conditions were as follows: initial time, 10% B; 1 min, 40% B; 3 min, 95% B; 5 min, 100% B, 5.1 min, 10% B; and re-equilibration to 7 min. The column was kept at 35°C. The injection volume was 10 *μ*L.

The compound was ionized in an electrospray ionization (ESI) instrument operated in positive mode. Capillary voltage was set at 3.5 kV. The sheath gas and aux gas were nitrogen at the pressures of 45 and 10 arb, respectively. The collision gas was argon at the vacuum pressure of 1.5 mTorr. Desolvation and source temperatures were set at 300°C and 350°C, respectively. To ensure high specificity and sensitivity, two or three of the most abundant precursor → fragment ion transitions (selected reaction monitoring, SRM) were observed for each analyte. SRM transitions, collision energies, and fragmentor for each analyte are given in Table S1.

### 2.4. Stir Bar Preparation

The glass capillary (2 mm diameter, 100 mm length) was cut into 20 mm long substrate. Then, a 16 mm iron rod was inserted in the substrate, and both of its ends were sealed by flame to generate a stir bar (20 mm × 2 mm). The pretreatment of the stir bar was carried out by successively placing it into 1 mol/L sodium hydroxide for 10 h and 0.1 mol/L hydrochloric acid for 1 h. Then, the stir bar was dried in an oven at 150°C for 1 h and immersed in a 30% (v/v) KH-570 solution in acetone for 3 h. Then, it was pulled out and cleaned with methanol.

Approximately 139.0 mg clenbuterol and 0.20 mL MAA were dissolved in 5 mL acetonitrile. The solution was mixed thoroughly and incubated overnight at room temperature. Then, 1.60 mL EGDMA and 20.0 mg AIBN were added, and the mixture was degassed by an ultrasonic device for 5 min. Then, a silylated stir bar was inserted into a glass tube, and 0.4 mL of solution was transferred into the tube. The glass tube was purged with nitrogen for 3 min and sealed by flame. The polymerization was accomplished in a water bath at 70°C for 24 h. The coating procedure was repeated two times. Then, the stir bar was pulled out by peeling off the outside glass tube and washed with 10% (v/v) acetic solution in methanol to remove the template molecules. The nonimprinted polymer-coated stir bar was prepared under the same conditions but without the addition of template clenbuterol in the synthesis.

### 2.5. Extraction Experiment

A round-bottom flask was used to conduct an extraction experiment in order to reduce the wear of the stir bar. The schematic diagram for the experiment is shown in Figure S1. The stir bar was immersed in solution and stirred at 500 rpm for 60 min at room temperature. Subsequently, the stir bar was removed from the sample solution, inserted in a 200 *μ*L glass vial with a conical insert, and desorbed with 100 *μ*L 10% (v/v) acid in water solution by an ultrasonic bath for 10 min. Then, 10 *μ*L desorption solution was injected into HPLC-MS/MS for analysis.

### 2.6. Sample Preparation

Samples of pork and liver were purchased from a local supermarket. A total of 2.0 g of each homogenized sample was spiked with *β*-agonist mixed standard solution. The spiked sample was extracted with 10 mL 2% (v/v) trichloroacetic acid solution in an ultrasonic bath for 10 min. The extraction procedure was repeated twice. The combined extracted solution was centrifuged at 5000 rpm for 5 min, and the pH of the supernatant was adjusted to 9-10 by 1 mol/L sodium hydroxide. Then, the solution was centrifuged again, and the supernatant was transferred to another Teflon centrifugal tube and extracted by 10 mL isopropyl alcohol/ethyl acetate (60 : 40, v/v) two times. The organic layers were combined and dried with nitrogen steam. Then, 5 mL toluene was added to dissolve the residual for SBSE.

## 3. Results and Discussion

### 3.1. Preparation of the Stir Bar

**Figure 1:**
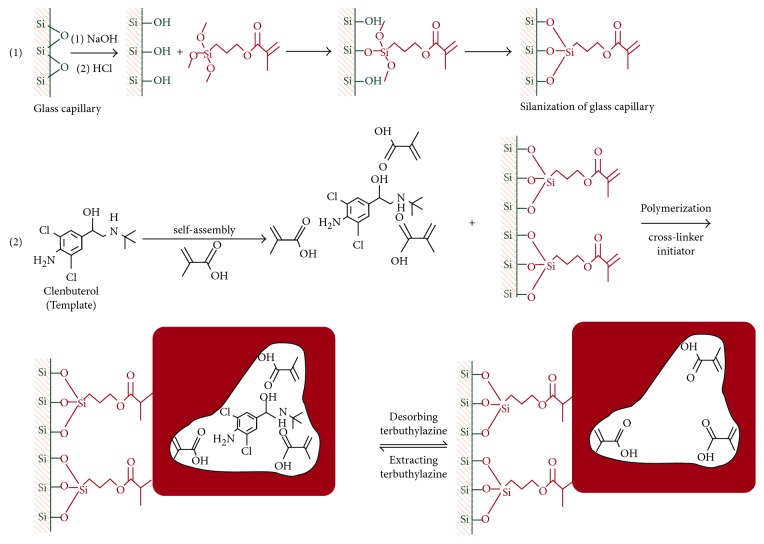
Schematic of preparation of molecularly imprinted stir bar.

The pretreatment and silanization of the glass bar were carried out to immobilize the MIP coatings on the bar. Soaking of the glass bar in alkaline solution is performed in order to expose the maximum number of silanol groups on the surface to promote silylation with the silane coupling agent. Thus, double bonds were present in the molecular structure, forming an active site for the MIP coating. After the overnight self-assembled polymerization of the template molecule and functional monomer through intermolecular hydrogen bonding interactions, the cross-linking agent and initiator were added to the prepolymerization solution, and thus, robust coatings were formed on the glass bar through thermal initiation polymerization. Then, the cavity structure with high selectivity for a target molecule was formed after eluting the template molecule by breaking the hydrogen bonding interaction between the functional monomer and template. The preparation schematic is illustrated in Figure 1.

The experimental parameters of MIP coating preparation such as the polymerization reagents and their proportion, temperature, and time were optimized. The experiment finally confirmed that the optimum mole ratio of template (clenbuterol), functional monomer (MAA), and cross-linker (EGDMA) was 1 : 4 : 16. The coatings of the MIP-coated stir bars prepared under optimum conditions were uniform and compact with a certain thickness (as shown in Figure S2).

### 3.2. Characterization of MIP Coatings

**Figure 2:**
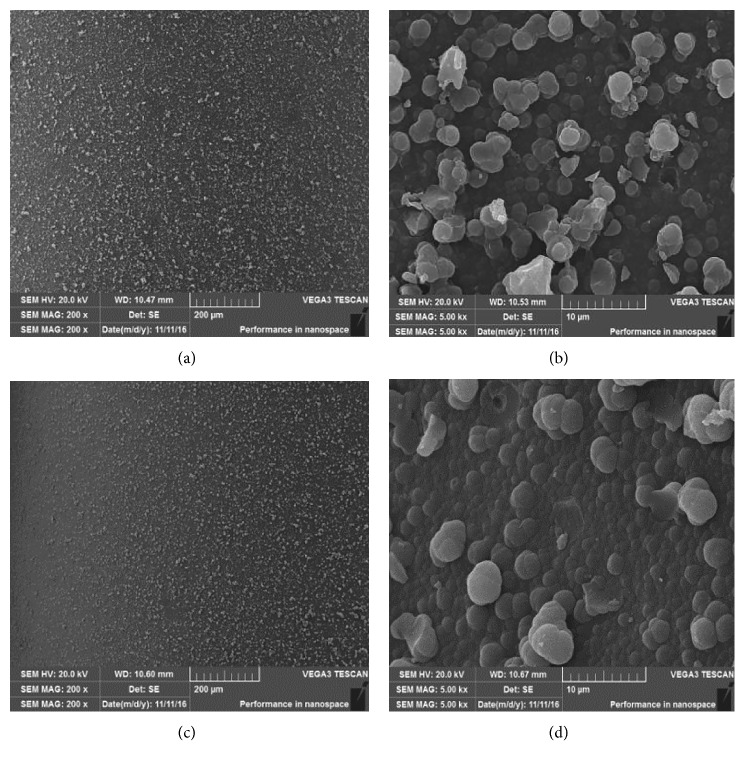
Scanning electron micrographs of (a) MIP-coated stir bar with the magnifications of 200x; (b) MIP-coated stir bar with the magnifications of 5000x; (c) NIP-coated stir bar with the magnifications of 200x; (d) NIP-coated stir bar with the magnifications of 5000x.

The morphological structure of the stir bar coating surface was characterized by scanning electron microscopy.  Figure 2 shows the surface structure of NIP coatings and MIP coatings under the magnification of 200x and 5000x. It is obvious that both MIP coatings and NIP coatings show homogeneous surfaces (Figures 2(a) and 2(c)), whereas the MIP coating exhibited a more porous structure than the NIP coating (Figures 2(b) and 2(d)). This is because the porous surface of the MIP coatings was more beneficial for analyte adsorption and desorption.

**Figure 3:**
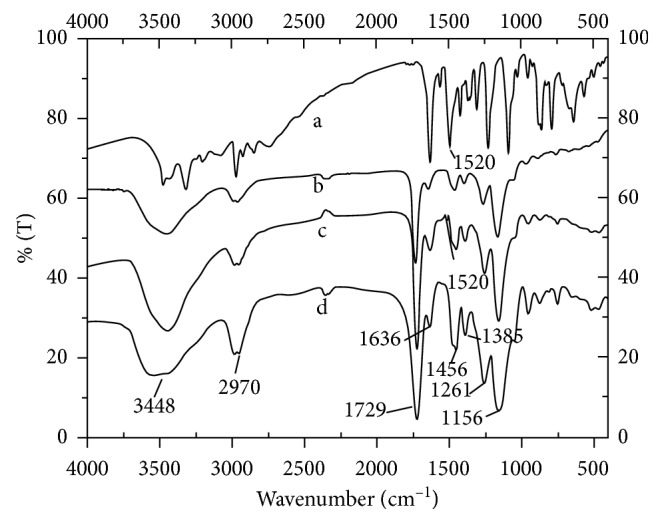
FT-IR spectra of clenbuterol molecularly imprinted polymer coatings. (a) Clenbuterol, (b) NIP coating, and (c) MIP coating before eluting template; (d) MIP coating after eluting template clenbuterol.

The molecular type and structure of the stir bar coatings were investigated by infrared spectroscopy. As shown in Figure 3, the absorption peaks of the NIP coating (Figure 3), MIP coating before eluting template (Figure 3), and MIP coating after eluting template (Figure 3) show no obvious differences except for the peak at 1520 cm^−1^, which was only present in the spectra of template (Figure 3) and MIP coating before eluting template (Figure 3). The absorption peak at 1520 cm^−1^ was derived from a C = C stretching vibration in the benzene ring of the template (clenbuterol). Therefore, it was reasonable to conclude that the template (clenbuterol) did not participate in the polymerization but only interacted with the functional monomer by hydrogen bonding.

### 3.3. Investigation of the Extraction Capability and Selectivity

#### 3.3.1. Extraction Capability

**Figure 4:**
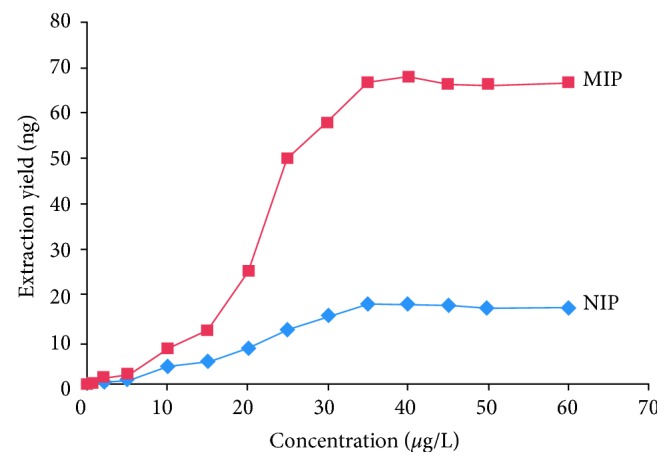
Extraction yield curves of MIP- and NIP-coated stir bars to various clenbuterol concentrations.

The adsorption capacity of the MIP-coated stir bar was studied with various clenbuterol standard solutions in toluene through static adsorption experiments. The NIP-coated stir bar was used for comparison. The extraction solution volume was 5 mL. The extraction time and desorption time were 60 and 10 min, respectively. As shown in Figure 4, the extraction amount of both the MIP-coated stir bar and NIP-coated stir bar increased with increasing clenbuterol concentration in the 1.0–60.0 *μ*g/L range, and the extraction amounts were balanced over the concentration of 35.0 *μ*g/L. The saturated adsorption amounts of the MIP coating and NIP coating were 67.0 and 17.8 ng, respectively. The adsorption amount of the MIP coating was approximately 3.76 times higher than that of the NIP coating. The excellent adsorption capacity of the MIP coating could be attributed to the molecular imprinting function of the clenbuterol template. The cavities of the MIP coating have a special affinity to the template, while no such cavities were present in the NIP coating that interacts with the template by nonspecific sorption only.

#### 3.3.2. Selectivity Evaluation

**Figure 5:**
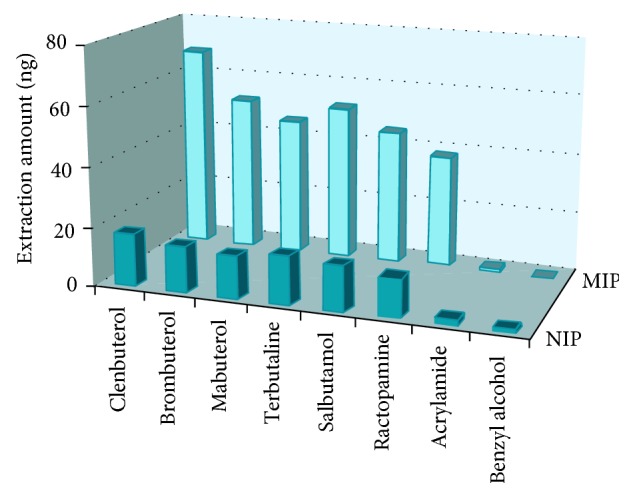
Extraction amount of clenbuterol and its structural analogues and reference compounds with MIP and NIP.

Clenbuterol and its structural analogues including ractopamine, salbutamol, mabuterol, terbutaline, and brombuterol were applied to extraction evaluation. Two reference compounds, namely, benzyl-alcohol and acrylamide, were used for comparison. All analytes were prepared individually with toluene to avoid competitive adsorption. The concentrations of all analytes were 10 *μ*g/L, and the extraction time was 60 min. As shown in Figure 5, the extraction amounts of the MIP coatings for clenbuterol, salbutamol, ractopamine, mabuterol, terbutaline, and brombuterol were 3.8, 2.9, 3.1, 3.5, 3.3, and 3.2 times higher, respectively, than those of the NIP coatings. By contrast, there was hardly any sorption of both kinds of coatings for benzyl-alcohol and acrylamide, which show different characteristics and structure. The selectivity of the MIP coating was closely related to the shape and size of the cavity, and the strength of interaction between the target molecules and the binding sites [39]. The steric shape complementary between the analytes and the recognition sites of MIPs plays a key role in the molecular recognition process. As a result, the MIP-coated stir bars exhibited higher selectivity toward the template of clenbuterol than its structural analogues. The analogues of salbutamol, mabuterol, terbutaline, and brombuterol with molecular shape and size similar to the template showed better imprinted effect than ractopamine, which had a little different molecular structure and functional groups. The reference compounds of benzyl-alcohol and acrylamide with less similarity with the template could hardly be extracted by both coatings.

### 3.4. Investigation of SBSE Conditions

To optimize the SBSE conditions, factors that affected the extraction results, such as solvent, time, stirring speed, and temperature, were also investigated in this work. The clenbuterol template was dissolved in the acetone, chloroform, acetonitrile, methanol, and toluene solvents to prepare individual solutions with a concentration of 10 *μ*g/L. The effect of extraction solvents on the extraction capacity was investigated. As shown in Figure S3, the maximum extraction amount was attained in the case of toluene solvent. The above results indicate that the primary driving forces behind the rebinding process, namely, the hydrophobic interaction and hydrogen bonding, were strongly related to the polarity of the extraction solvents. The hydrogen bonding was liable to be formed in noncovalently imprinted polymers, and higher adsorptive capacity was obtained in the solvents with weaker polarity. Thus, the matrix effect of water should be removed, which influenced the absorption efficiency of MIP-coated stir bars. The water, methanol, methanol-acetic acid (9 : 1, v/v), and water-acetic acid (9 : 1, v/v) solvents were selected for the investigation of the solvent effect on the desorption. The desorbed amounts were 26.5, 25.6, 27.5, and 30.5 ng, respectively. Therefore, water-acetic acid (9 : 1, v/v) was chosen as the best desorption solvent.

The extraction and desorption amounts increased with increasing time, and the times at which each reached equilibrium were 60 min and 10 min, respectively (Figures S4 and S5). Therefore, 60 min and 10 min were adopted for extraction and desorption procedures.

The stirring speed varying from 0 to 800 rpm was investigated. The results indicate that the extraction amounts increased with the stirring speed and the maximum extraction amount was obtained at 300 rpm. Beyond 300 rpm, no obvious increase was attained. Thus, 300 rpm was chosen as the optimal stirring speed. In addition, the change in the extraction temperature exerted no obvious influence on the extraction amounts. For convenience, the adsorption experiments were carried out at room temperature.

### 3.5. Durability Investigation

To investigate the stability of the coating, the MIP-coated stir bar was immersed into conventional organic solvents, such as toluene, acetonitrile, methanol, *n*-hexane, ethanol, chloroform, ethyl acetate, dimethylsulfoxide, and tetrahydrofuran. After stirring for 24 h, no decomposition and exfoliation were obtained, indicating that the coatings of MIP-coated stir bar possessed excellent antisolvent fastness properties. The MIP-coated stir bar could be reused after the analyte was removed by methanol-acetic acid (9 : 1, v/v). As the extraction was performed in a round-bottom flask instead of a flat-bottom bottle, the wearing of the stir bar during the stirring has been dramatically reduced. The coating was kept intact, and no decrease in the extraction efficiency was obtained after extraction for 60 times.

### 3.6. Method Validation

Assay of clenbuterol and its structural analogues by MIP-coated SBSE coupled with HPLC-MS/MS was performed. The blank pork and liver samples were spiked with six *β*-agonists and taken for analysis. The linear dynamic ranges, correlation coefficients, recoveries, LODs, LOQs, and reproducibility under the optimized experimental conditions are listed in Table 1. The linear range was determined to be 0.50–35 *μ*g/kg for clenbuterol and 1.0–35 *μ*g/kg for ractopamine, mabuterol, brombuterol, or terbutaline. Good linearity was obtained for six *β*-agonists with correlation coefficients (*R* ^2^) higher than 0.994. The limits of detection (LODs, signal-to-noise ratio equal to 3) and limits of quantification (LOQs, signal-to-noise ratio equal to 10) were in the ranges of 0.05–0.15 *μ*g/kg and 0.10–0.30 *μ*g/kg, respectively. The recoveries for the spiked pork and liver samples ranged from 75.8% to 97.9%. The RSD for intraday and interday assays of six *β*-agonists varied from 2.6 to 5.3% (*n*=5).

### 3.7. Application of the MIP-Coated Stir Bar

**Figure 6:**
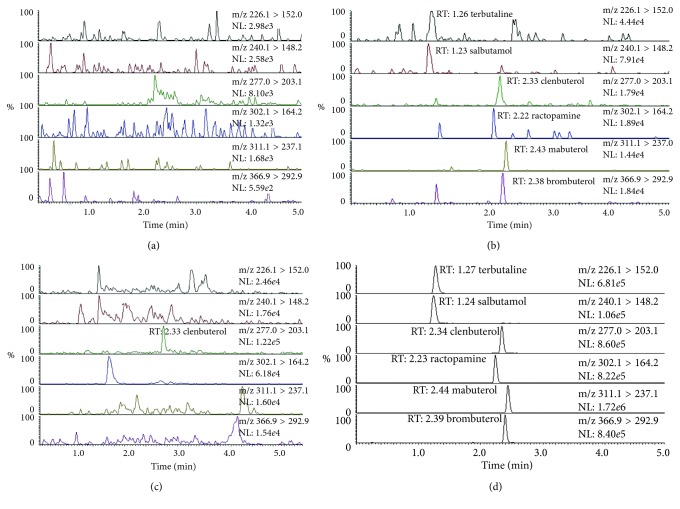
Multiple reaction monitoring (MRM) chromatograms showing the transitions corresponding to the quantifier ions of six *β*-agonists. (a) The blank pork sample; (b) the pork sample spiked with each *β*-agonist at LOD level; (c) the real pork sample; (d) the pork sample spiked with each *β*-agonist at 5.0 *μ*g/kg.

To validate the selectivity of the method for assay of complex sample matrix, the blank pork samples were spiked with six *β*-agonists at LOD level and 5.0 *μ*g/kg. Furthermore, the real sample collected from the local market was analyzed by the proposed method. The multiple reaction monitoring (MRM) chromatograms showing the transitions corresponding to the quantifier ions of six *β*-agonists are depicted in Figure 6. *β*-Agonists could not be quantitatively assayed in the blank pork sample (Figure 6(a)). However, the six *β*-agonists were accurately analyzed based on MIP-SBSE (Figure 6(b)). Using the MIP-coated stir bar, the matrix interference has been largely eliminated. As shown in Figure 6(c), the peak at the retention time of 2.33 min was detected in the channel of *m/z* 277.0/203.1, suggesting that clenbuterol might exist in the pork sample. The extracts were subjected to further analysis using accurate mass AB Sciex Triple Quad 5500 LC/MS/MS in MS/MS/MS scan mode, and the content of clenbuterol in the positive pork sample was determined to be 1.31 *μ*g/kg. The analysis of the pork sample spiked with each *β*-agonist at 5.0 *μ*g/kg is shown in Figure 6(d). The appearance time for the six *β*-agonists was within 3 min, and the total analysis time for each specimen was only 5 min. The performance comparison of the proposed method based on MIP-SBSE and other analytical methods for the determination of *β*-agonists is shown in Table 2. Our work was advantageous in terms of detection throughput, sensitivity, robustness, and reproducibility. The proposed method has been successfully applied to the determination of trace *β*-agonists in foodstuff of animal origin with improved enrichment ratio of target analytes and reduced sample matrix effect.

## 4. Conclusions

A new clenbuterol MIP-coated stir bar for the assay of *β*-agonists with similar structures was constructed. The new coatings possessed homogeneous and porous morphology and exhibited excellent extraction capability and selectivity for six *β*-agonists. The MIP-coated SBSE coupled with HPLC-MS/MS has been successfully applied to the analysis of pork and liver samples spiked with *β*-agonists, and the recoveries ranged from 75.8 to 97.9%. In comparison with other analytical methods, our work possesses significant advantages of high throughput, high sensitivity, high efficiency, and low detection limit. The approach described herein may be extended to the preparation of a series of selective MIP-coated stir bars with appropriate template molecules for complex sample analysis.

## Figures and Tables

**Table 1:** Linear dynamic range, correlation coefficients, LODs and LOQs, average recoveries, and interday and intraday precisions achieved for the six *β*-agonists.

Compounds	Linear range (*μ*g/L)	*R* ^2^	LOD^a^ (*μ*g/L)	LOQ^b^ (*μ*g/L)	Recovery (%)			Interday assay^c^ (RSD %, *n*=5)	Intraday assay^c^ (RSD %, *n*=5)
					Spiked level (*μ*g/kg)	Pork	Liver		
Clenbuterol	0.5–35	0.9991	0.05	0.10	1.0	85.0	87.2	3.9	4.2
					5.0	86.5	97.9		
Salbutamol	1–35	0.9981	0.10	0.25	1.0	85.3	84.5	4.5	5.1
					5.0	76.5	88.2		
Ractopamine	1–35	0.9952	0.15	0.35	1.0	75.8	80.2	5.1	5.3
					5.0	83.6	72.6		
Mabuterol	1–35	0.9978	0.10	0.25	1.0	81.2	80.8	4.4	2.6
					5.0	85.3	84.9		
Brombuterol	1–35	0.9936	0.10	0.25	1.0	84.5	82.6	4.6	3.8
					5.0	85.8	85.6		
Terbutaline	1–35	0.9957	0.15	0.30	1.0	84.3	83.1	4.9	4.1
					5.0	85.4	85.2		

^a^S/N = 3; ^b^S/N = 10; ^c^assays at 5.0 *μ*g/kg level.

**Table 2:** Comparison of the proposed method based on MIP-SBSE with other analytical methods for the determination of *β*-agonists in pork sample.

Sample pretreatment	Analytical method	Target analyte number	Matrix	Recovery (%)	Precision RSD (%)	LOD (*μ*g/kg)	References
MIP-SBSE	HPLC	3	Pork	83.7–92.3	2.9–8.1	0.10–0.21	[40]
SPE	GC-MS	4	Pork	75.5–89.6	2.5–4.2	0.4–0.7	[41]
SPE	UPLC-MS/MS	4	Pork	65.5–109.1	6.6–15.4	0.1	[42]
QuEChERS	LC-MS/MS	10	Meat	73.7–103.5	2.7–15.3	0.2–0.9	[43]
SPE	HPLC-MS/MS	3	Pork	79.25–112.82	7.14%–17.80	0.13–0.15	[44]
MIP-SBSE	HPLC-MS/MS	6	Pork	75.8–97.9	2.6–5.3	0.05–0.15	This work

## Data Availability

The data used to support the findings of this study are available from the corresponding author upon request.

## Supplementary Materials

Supplementary Materials Figure S1: schematic diagram of homemade device. Table S1: precursor ion, product ions, optimal collision voltage, and tube lens for each MS/MS transition of the analyte. Figure S2: photograph of MIP-coated stir bar. Figure S3: the extraction yield of CL with different extraction solvents (*n*=3). Figure S4: sorption kinetics of the MIP-coated stir bar for CL solution. Figure S5: desorption kinetics of the MIP-coated-stir bar for CL solution.

## References

[1] Guggenbuhl P. (2010). Evaluation of β2-adrenergic agonists repartitioning effects in the rat by a non-destructive method. *Journal of Animal Physiology and Animal Nutrition*.

[2] Johnson B. J., Smith S. B., Chung K. Y. (2014). Historical overview of the effect of beta-adrenergic agonists on beef cattle production. *Asian-Australasian Journal of Animal Sciences*.

[3] Brambilla G., Cenci T., Franconi F. (2000). Clinical and pharmacological profile in a clenbuterol epidemic poisoning of contaminated beef meat in Italy. *Toxicology Letters*.

[4] Barbosa J., Cruz C., Martins J. (2005). Food poisoning by clenbuterol in Portugal. *Food Additives and Contaminants*.

[5] Beucher L., Dervilly-Pinel G., Prévost S., Monteau F., Le Bizec B. (2015). Determination of a large set of beta-adrenergic agonists in animal matrices based on ion mobility and mass separations. *Analytical Chemistry*.

[6] Cooper A. D., Shepherd M. J. (1996). Evaluation of a novel immunoaffinity phase for the purification of cattle liver extracts prior to high-performance liquid chromatographic determination of β-agonists. *Food and Agricultural Immunology*.

[7] Jiang D., Cao B., Wang M. (2017). Development of a highly sensitive and specific monoclonal antibody based enzyme-linked immunosorbent assay for the detection of a new beta-agonist, phenylethanolamine A, in food samples. *Journal of the Science of Food and Agriculture*.

[8] Liu H., Gan N., Chen Y. (2016). Novel method for the rapid and specific extraction of multiple β2-agonist residues in food by tailor-made Monolith-MIPs extraction disks and detection by gas chromatography with mass spectrometry. *Journal of Separation Science*.

[9] Zhang M. Z., Wang M.-Z., Chen Z.-L. (2009). Development of a colloidal gold-based lateral-flow immunoassay for the rapid simultaneous detection of clenbuterol and ractopamine in swine urine. *Analytical and Bioanalytical Chemistry*.

[10] Zhu Y., Xie S., Chen D. (2016). Targeted analysis and determination of beta-agonists, hormones, glucocorticoid and psychiatric drugs in feed by liquid chromatography with electrospray ionization tandem mass spectrometry. *Journal of Separation Science*.

[11] Du H., Chu Y., Yang H. (2016). Sensitive and specific detection of a new β-agonist brombuterol in tissue and feed samples by a competitive polyclonal antibody based ELISA. *Analytical Methods*.

[12] Liu H., Lin X., Lin T., Zhang Y., Luo Y., Li Q. (2016). Magnetic molecularly imprinted polymers for the determination of β-agonist residues in milk by ultra high performance liquid chromatography with tandem mass spectrometry. *Journal of Separation Science*.

[13] Zhang Z., Yan H., Cui F. (2016). Analysis of multiple β-agonist and β-blocker residues in porcine muscle using improved QuEChERS Method and UHPLC-LTQ Qrbitrap mass spectrometry. *Food Analytical Methods*.

[14] Wang X., Liu Y., Su Y. (2014). High-throughput screening and confirmation of 22 banned veterinary drugs in feedstuffs using LC-MS/MS and high-resolution Orbitrap mass spectrometry. *Journal of Agricultural and Food Chemistry*.

[15] Couchman L., Morgan P. E. (2011). LC-MS in analytical toxicology: some practical considerations. *Biomedical Chromatography*.

[16] Nakamura M. (2011). Analyses of benzodiazepines and their metabolites in various biological matrices by LC-MS(/MS). *Biomedical Chromatography*.

[17] Henze M. K., Opfermann G., Spahn-Langguth H., Schänzer W. (2001). Screening of β-2 agonists and confirmation of fenoterol, orciprenaline, reproterol and terbutaline with gas chromatography-mass spectrometry as tetrahydroisoquinoline derivatives. *Journal of Chromatography B: Biomedical Sciences and Applications*.

[18] Sharafi K., Fattahi N., Hossein Mahvi A., Pirsaheb M., Azizzadeh N., Noori M. (2015). Trace analysis of some organophosphorus pesticides in rice samples using ultrasound-assisted dispersive liquid-liquid microextraction and high-performance liquid chromatography. *Journal of Separation Science*.

[19] Hermida L., Rodríguez R., Lazo L. (2002). A recombinant envelope protein from Dengue virus purified by IMAC is bioequivalent with its immune-affinity chromatography purified counterpart. *Journal of Biotechnology*.

[20] Cai Y., Jiang G., Liu J., Zhou Q. (2003). Multiwalled carbon nanotubes as a solid-phase extraction adsorbent for the determination of bisphenol A, 4-n-nonylphenol, and 4-tert-octylphenol. *Analytical Chemistry*.

[21] Lindsey M. E., Meyer T. M., Thurman E. M. (2001). Analysis of trace levels of sulfonamide and tetracycline antimicrobials in groundwater and surface water using solid-phase extraction and liquid chromatography/mass spectrometry. *Analytical Chemistry*.

[22] Mullett W. M., Paul Martin A., Pawliszyn J. (2001). In-tube molecularly imprinted polymer solid-phase microextraction for the selective determination of propranolol. *Analytical Chemistry*.

[23] Ouyang G., Vuckovic D., Pawliszyn J. (2011). Nondestructive sampling of living systems using in vivo solid-phase microextraction. *Chemical Reviews*.

[24] Baltussen E., Sandra P., David F., Cramers C. (2015). Stir bar sorptive extraction (SBSE), a novel extraction technique for aqueous samples: theory and principles. *Journal of Microcolumn Separations*.

[25] Kawaguchi M., Ito R., Saito K., Nakazawa H. (2006). Novel stir bar sorptive extraction methods for environmental and biomedical analysis. *Journal of Pharmaceutical and Biomedical Analysis*.

[26] Xia Y., McGuffey J. E., Bhattacharyya S. (2005). Analysis of the tobacco-specific nitrosamine 4-(methylnitrosamino)-1-(3-pyridyl)-1-butanol in urine by extraction on a molecularly imprinted polymer column and liquid chromatography/atmospheric pressure ionization tandem mass spectrometry. *Analytical Chemistry*.

[27] Masqué N., Marcé R. M., Borrull F., Cormack P. A. G., Sherrington D. C. (2000). Synthesis and evaluation of a molecularly imprinted polymer for selective on-line solid-phase extraction of 4-nitrophenol from environmental water. *Analytical Chemistry*.

[28] Lachová M., Lehotay J., Skacani I., Jozef C. (2010). Study of selectivity of molecularly imprinted polymers prepared under different conditions. *Journal of Chromatographic Science*.

[29] Tamayo F. G., Turiel E., Martin-Esteban A. (2007). Molecularly imprinted polymers for solid-phase extraction and solid-phase microextraction: recent developments and future trends. *Journal of Chromatography A*.

[30] Chen C., Yang L., Jie Z. (2011). Trace bensulfuron-methyl analysis in tap water, soil, and soybean samples by a combination of molecularly imprinted stir bar sorption extraction and HPLC-UV. *Journal of Applied Polymer Science*.

[31] Díazálvarez M., Turiel E., Martínesteban A. (2016). Molecularly imprinted polymer monolith containing magnetic nanoparticles for the stir-bar sorptive extraction of triazines from environmental soil samples. *Journal of Chromatography A*.

[32] Prasad B. B., Srivastava A., Tiwari M. P. (2013). Highly selective and sensitive analysis of dopamine by molecularly imprinted stir bar sorptive extraction technique coupled with complementary molecularly imprinted polymer sensor. *Journal of Colloid and Interface Science*.

[33] Wu X., Liu J., Wu J. (2012). Molecular imprinting-based micro-stir bar sorptive extraction for specific analysis of glibenclamide in herbal dietary supplements. *Journal of Separation Science*.

[34] Zhong Q., Hu Y., Hu Y., Li G. (2012). Online desorption of molecularly imprinted stir bar sorptive extraction coupled to high performance liquid chromatography for the trace analysis of triazines in rice. *Journal of Separation Science*.

[35] Zhu L., Xu G., Wei F., Yang J., Hu Q. (2015). Determination of melamine in powdered milk by molecularly imprinted stir bar sorptive extraction coupled with HPLC. *Journal of Colloid and Interface Science*.

[36] Zhu X., Cai J., Yang J., Su Q., Gao Y. (2006). Films coated with molecular imprinted polymers for the selective stir bar sorption extraction of monocrotophos. *Journal of Chromatography A*.

[37] Xu Z., Song C., Hu Y., Li G. (2011). Molecularly imprinted stir bar sorptive extraction coupled with high performance liquid chromatography for trace analysis of sulfa drugs in complex samples. *Talanta*.

[38] Xu Z., Yang Z., Liu Z. (2014). Development of dual-templates molecularly imprinted stir bar sorptive extraction and its application for the analysis of environmental estrogens in water and plastic samples. *Journal of Chromatography A*.

[39] Liu R., Li X., Li Y., Jin P., Qin W., Qi J. (2010). Effective removal of rhodamine B from contaminated water using non-covalent imprinted microspheres designed by computational approach. *Biosensors and Bioelectronics*.

[40] Xu Z., Hu Y., Hu Y., Li G. (2010). Investigation of ractopamine molecularly imprinted stir bar sorptive extraction and its application for trace analysis of β2-agonists in complex samples. *Journal of Chromatography A*.

[41] Wang Q. S., Sun W. M., Liu J. R. (2015). Determination of four clenbuterol residues in pork by solid phase extraction and gas chromatography-mass spectrometry. *Occupation and Health*.

[42] Wang T. Z. (2016). Simultaneous determination of 4 kinds of β-agonist residues in pork by ultra performance liquid chromatography-electrospray tandem mass spectrometry. *Journal of Food Safety and Quality*.

[43] Xiong L., Gao Y.-Q., Li W.-H., Yang X.-L., Shimo S. P. (2015). Simple and sensitive monitoring of β2-agonist residues in meat by liquid chromatography-tandem mass spectrometry using a QuEChERS with preconcentration as the sample treatment. *Meat Science*.

[44] Zhang R. Y. (2017). Determination of 3 kinds of β-agonists residues in pork by high performance liquid chromatography-tandem mass spectrometry. *Journal of Food Safety and Quality*.

