# Supplementary material for: Determination of β-Agonist Residues in Animal-Derived Food by a Liquid Chromatography-Tandem Mass Spectrometric Method Combined with Molecularly Imprinted Stir Bar Sorptive Extraction
Source: J Anal Methods Chem. 2018 Jun 25;2018:9053561. doi: 10.1155/2018/9053561 (PMC6036788; doi:10.1155/2018/9053561)
Supplement: Supplementary Materials — Figure S1: schematic diagram of homemade device. Table S1: precursor ion, product ions, optimal collision voltage, and tube lens for each MS/MS transition of the analyte. Figure S2: photograph of MIP-coated stir bar. Figure S3: the extraction yield of CL with different extraction solvents (n=3). Figure S4: sorption kinetics of the MIP-coated stir bar for CL solution. Figure S5: desorption kinetics of the MIP-coated-stir bar for CL solution. [file 9053561.f1.pdf]

Supplementary Material for determination of β-agonist residues in animal-derived food by a liquid chromatography-tandem mass spectrometric method combined with molecularly imprinted stir bar sorptive extraction

Jiwang Tang [1,2,], Jianxiu Wang [1], Shuyun Shi [1], Shengqiang Dong [1], Liejiang Yuan [2]

[1]College of Chemistry and Chemical Engineering, Central South University, Changsha, 410083, China
[2]Hunan Testing institute Product and commodity Supervison, Changsha, 422700, China

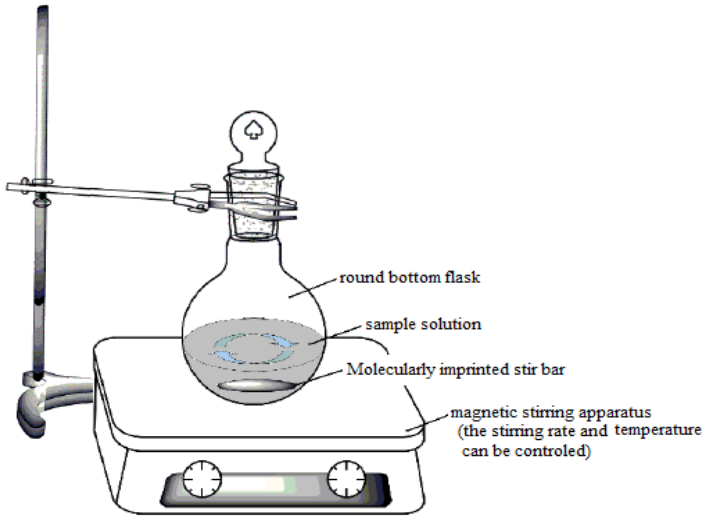

Fig. S1. Schematic diagram of homemade device.

Table S1 Precursor ion, product ions, optimal collision voltage and tube lens for each MS/MS transition of the analyte.

analyte	precursor ion [M H]+	product ions	collision energy (eV)	tube lens
clenbuterol	277.0	132.1	24	93
	277.0	*203.1	14	93
salbutamol	240.1	121.2	33	107
	240.1	*148.2	17	107
ractopamine	302.1	107.1	32	108
	302.1	*164.2	15	108
mabuterol	311.1	*237.1	14	100
	311.1	293.0	16	100
brombuterol	366.9	*292.9	19	117
	366.9	349.0	25	117
terbutaline	226.1	*152.0	13	98
	226.1	125.1	15	98

*quantitative ion

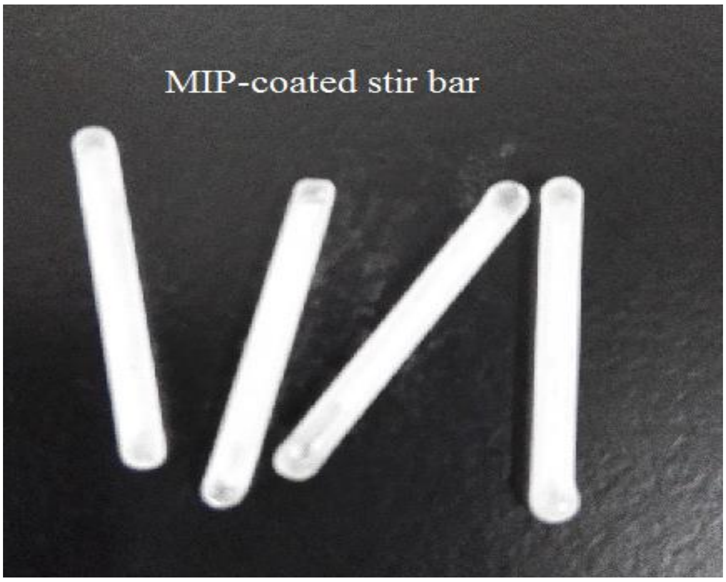

Fig. S2. Photograph of MIP-coated stir bar.

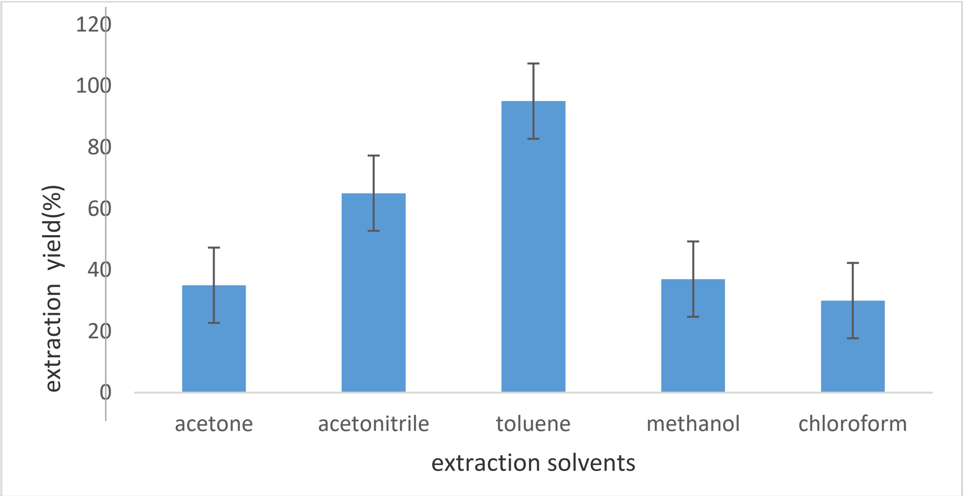

Fig. S3. The extraction yield of CL with different extraction solvents (n = 3).

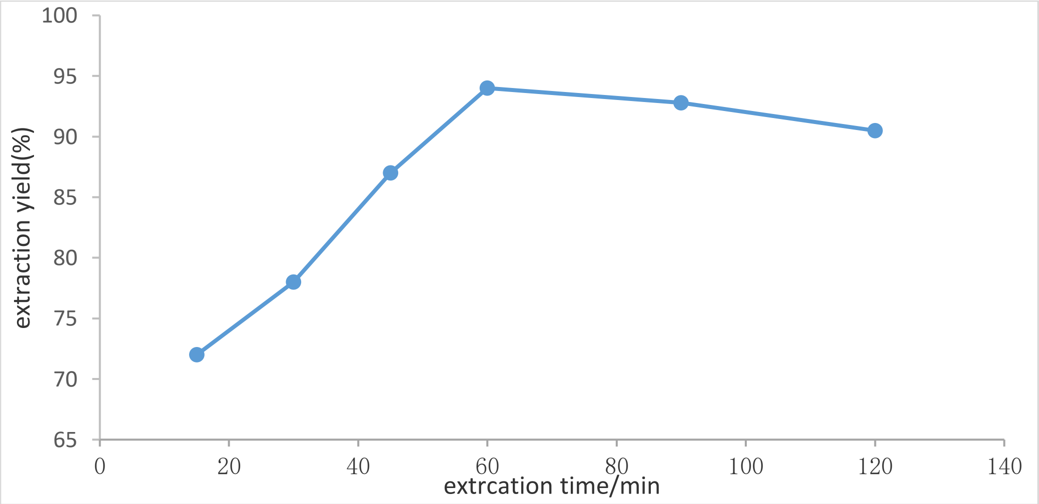

Fig. S4. Sorption kinetics of the MIPs-stir bar for CL solution.

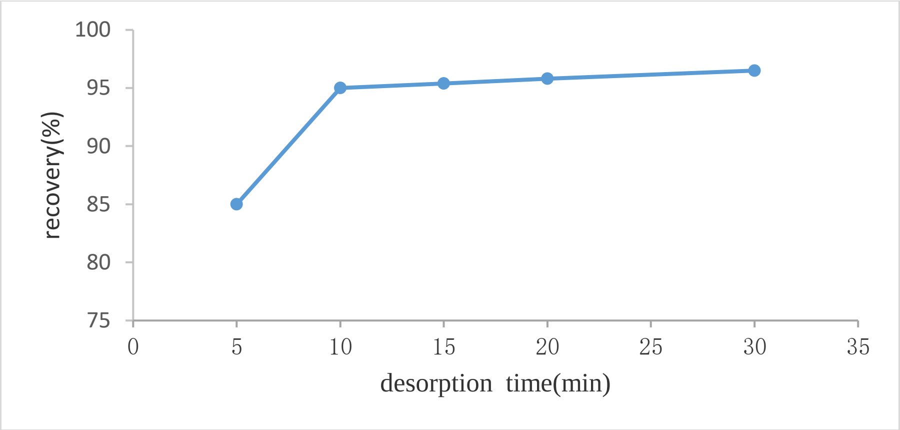

Fig. S5. Desorption kinetics of the MIPs-stir bar for CL solution.
